# The rise of diarrheal illnesses in the children of Pakistan amidst COVID‐19: A narrative review

**DOI:** 10.1002/hsr2.1043

**Published:** 2023-01-03

**Authors:** Zainab Syyeda Rahmat, Amraha Zubair, Ikran Abdi, Narmil Humayun, Fatima Arshad, Mohammad Yasir Essar

**Affiliations:** ^1^ Faculty of Medicine, Dow Medical College Dow University of Health Sciences Karachi Pakistan; ^2^ Faculty of Medicine, Sindh Medical College Jinnah Sindh Medical University Karachi Pakistan; ^3^ Department of Dentistry, Kabul University of Medical Sciences Kabul Afghanistan

**Keywords:** children, COVID‐19, diarrhea, Pakistan

## Abstract

**Background:**

In Pakistan, 74 children out of 1000 lose their lives annually due to diarrheal illness. This commentary addresses the contributing factors aggravating this growing dilemma and the effect of a simultaneous rise in COVID‐19 cases in a healthcare system ready to collapse, along with providing recommendations to alleviate the problems causing this spike in diarrheal cases.

**Methods:**

This narrative review has emphasized the causes of the spike in pediatric diarrheal illnesses in Pakistan as well as recommendations offered to lessen the burden by incorporating recent literature (*n* = 68). Tactics to tackle COVID‐19 alongside diarrheal illnesses were also included. Pakistan was chosen to be assessed due to its high burden of child and infant mortality due to preventable causes.

**Results:**

The provision of safe drinking water, the proper use of Integrated Management of Newborn and Childhood Illnesses (IMNCI), adequate awareness of the benefits of breastfeeding, the use of correct rehydration techniques such as Oral Rehydration Therapy, and the crucial implementation of the EPI vaccination schedule can curb this increase in diarrheal cases.

**Conclusion:**

The increased prevalence of diarrheal diseases amongst the pediatric population of Pakistan can be attributed largely due to unsanitary drinking water. Emphasis must be put in the provision of safe drinking water. During the first years of life, children can be kept safe from deadly pathogens just by adequate breastfeeding. IMNCI and EPI must also be properly implemented.

## 1 INTRODUCTION

Diarrhea is defined as loose, watery stools that occur three or more times per day.^1^ It can be categorized as acute, persistent, or chronic.^1^ Acute diarrhea can be divided into watery and bloody, both caused by different pathogens such as Vibrio Cholerae and Campylobacter, respectively.^2^ Amongst children, diarrhea is a common occurrence, and it is considered normal for young infants to have 3−10 loose stools daily.^3^ If the frequency rises to twice the number of normal stools, it is considered diarrhea for young infants. Although both treatable and preventable, diarrhea is the cause of death for 2195 children daily around the world, which is more than malaria, AIDS, and measles combined.^2^, ^4^

Worldwide, 1 in 9 child deaths are due to diarrheal illnesses and 801,000 children die every year from diarrhea.^4^ Diarrhea also reduces the absorption of vital nutrients, and is a leading contributor to malnutrition in children under 5.^2^, ^5^ In the early years of life, diarrheal diseases constitute 10‐80% of growth retardation worldwide.^6^ The predominant cause of acute diarrhea in children is viral infection with rotavirus being the most common cause, accounting for 40% of hospitalizations amongst children under 5.^3^, ^4^ Other causes include bacterial infections, lack of proper breastfeeding, contaminated water, antibiotic effects, and parasitic infections.^3^

In Pakistan, a country of over 225 million, 60% of infant and child deaths are caused by diarrhea.^7^, ^8^ Children under 5 comprise of only 15% of the population, yet make up 50% of the mortality rate.^9^ These statistics do not reflect the true situation due to a lack of a functioning disease surveillance system, as well as inadequate data on births and deaths.^10^ However, Pakistan has the highest ratio in Asia for infant mortality from diarrhea.^11^ The World Health Organization (WHO) has ranked Pakistan 23 in terms of childhood mortality caused by diarrheal illness, with almost 6.4 million cases of pediatric diarrhea annually.^12^ Recently, diarrheal cases in the pediatric population have been on the rise throughout the country, with over 2000 children being admitted in the city of Lahore in just one month's time.^13^ In the cities of Islamabad and Rawalpindi, hundreds of children are being admitted daily with bouts of acute viral diarrhea.^14^ The situation is mimicked in Karachi, where hospitals are witnessing a surge in diarrheal cases, particularly among children between 3 months and 5 years.^15^ In 2022, the province of Sindh is witnessing a surge in cholera cases, with 234 confirmed cases reported from January to May.^16^ Eighteen of the 27 deaths in Baluchistan were of children under 7 due to the cholera outbreak, as of May 2022.^17^ One way to curb this increase in diarrheal diseases especially in children is to establish educational programs throughout schools in Pakistan to enlighten the public about healthy safety measures, and the first step is to adhere correctly to a theory or model of health education.^18^

Worldwide, almost 88% of deaths associated with diarrhea were linked to unsafe water amongst other causes.^4^ The probability of diarrheal morbidity and mortality is higher among people with lower socioeconomic status and in circumstances of poor personal and substandard domestic hygiene conditions.^19^ The poverty rate in Pakistan has been on a continuous rise since the 90 s, with rates of over 32% in 1999 increasing to above 39% in 2020 as estimated by the World Bank.^20^, ^21^ Only 20% of the Pakistani population have access to clean water.^11^ Fifty percent of all diseases and 40% of all deaths recorded in Pakistan are due to utilization of polluted water.^11^ More than 66% of Pakistani households drink water contaminated with bacteria, resulting in rampant diarrheal illness amongst other diseases such as hepatitis.^22^ Contaminated water carries various pathogens causing diarrheal disease, including cholera, amoebiasis, gastroenteritis, giardiasis, and campylobacteriosis, to name a few.^23^

Another leading contributor to diarrhea amongst infants in Pakistan is the lack of proper breastfeeding. Babies who are breastfed are proven to have stronger immune systems and less diarrheal episodes and gastroenteritis, also known as stomach flu.^24^ However, only 37% of Pakistani women breastfeed their infants exclusively for 6 months, and lack of breastfeeding is correlated with a 165% increase in the frequency of diarrheal episodes amongst infants under 6 months.^10^, ^25^ In comparison to those exclusively breastfed, infants who were not breastfed are 15 times more likely to lose their lives due to diarrhea.^10^ Therefore, proper awareness and empowerment of mothers is crucial for the betterment of their children. Closing gender gaps along with providing economic opportunities for women's entrepreneurship may positively impact their children's lives.^26^

COVID‐19, first isolated from Wuhan, China in December of 2019, was declared a global pandemic in March 2020. Lockdowns imposed by governments throughout the world have led to drastic changes in peoples' lives, and food systems disrupted.^27^, ^28^ Two years and multiple variants later, cases are rising again in Pakistan, with the positivity ratio reaching the highest in 5 months.^29^ In a single day, the ratio reached 5.46% on July 13, 2022.^29^ Over 30,000 deaths have been recorded in Pakistan since 2021 due to COVID‐19.^30^ The pandemic is caused further strain on an exhausted healthcare system, and surges in diarrheal cases throughout the country may push the country's resources to its brink. Thus, this article offers insights into the trials faced by healthcare professionals and the country in eradicating the causes of diarrheal illnesses especially amongst children, while also battling a surge in COVID‐19 cases.

## 2 LITERATURE SEARCH

The covariates associated with diarrheal mortality have been classified as unsafe water, lower socioeconomic conditions, poor personal and substandard hygiene conditions, lack of breastfeeding and immunization according to the Expanded Program of Immunization (EPI), and standard management and treatment strategies. Our methods are coherent with the frameworks and models developed in the previously existing literature.^31^, ^32^, ^33^, ^34^

Database searches were performed (PubMed and Google Scholar) using the search terms (diarrhea or dysentery) and (children or infants) and (Pakistan) and (COVID‐19). Moreover, additional searches included (bottle‐fed or formula milk) AND (water‐borne diseases). Key terms such as (Rotavirus or *G. lamblia* or vibrio cholera or campylobacter), “Breastfeeding,” (ORS OR Oral Rehydration Therapy), (Expanded Program of Immunization OR EPI), (IMNCI OR Integrated Management of Childhood Illness) were also used.

Management of large epidemics requires a competent referral system, justice, and participation of the public alongside government. These measurements are required to lessen disease burden.^35^ Since the emergence of COVID‐19 in December 2019, the whole world remains in a state of turmoil. Strategies to increase isolation by quarantining is a useful model in regulating transmission and swift spread. As people began staying indoors, many educational institutes shut down, which closed the coping source of many students that have numerous personal and familial issues.^36^ Recorded data by the National Institute of Health in Pakistan has computed that the COVID‐19 outbreak entered Pakistan through international travelers.^37^ Lockdowns and restrictions due to COVID‐19 may have negatively affected self‐esteem, especially in the elderly population. Group logotherapy has been found to positively affect self‐esteem in the elderly.^38^

## 3 INCLUSION AND EXCLUSION CRITERIA

Articles were excluded if they were irrelevant, published before 2000, and in languages other than English. Moreover, articles pertaining to countries other than Pakistan were excluded, along with those that were not associated with the keywords.

Any study found in the literature search was included (reviews or original articles, such as observational or cross‐sectional studies). The studies meeting the childhood diarrhea definition of three or more watery or abnormally loose stools within the previous 24 h in a child younger than 5 years of age were taken into consideration.

## 4 DISCUSSION

There are several ways to combat deaths in infants caused by diarrheal illnesses. Amongst the most vital is vaccination against common diarrhea‐causing organisms. For example, Rotavirus is one of the most common causes of diarrheal related deaths in infants, which is why vaccines have been developed against it and are approved in almost 200 countries.^39^ Another important reason for urgency in combatting these infectious diseases is the overwhelming percentage of children inhabiting developing countries like Pakistan; developing countries are comprised of 85% of the world's young population.^40^ Pakistan being a third world nation faces its own set of challenges when it comes to achieving herd immunity against infectious agents. Along with being incapable of affording the correct number of vaccines to fulfill the country's requirements, it also lacks facilities to store and transport them to remote rural areas.^41^ Increasing resource depletion which is demonstrated by resource reduction is not just a problem in Pakistan, in fact, it is a worldwide phenomenon.^42^ Just as food products must be safe for consumer usage, the proper storage, transfer, and utilization of vaccines is crucial for health.^43^ Moreover, this dilemma is aggravated by hoarding of vaccines by developed countries of the world, automatically creating a shortage in chain supply.^44^ Studies have been carried out for creating vaccine equality among nations via vaccine empathy and diplomacy, which involves vaccine donations from the privileged nations to the needy.^45^ While vaccine empathy can help Pakistan achieve its goals of reducing death tolls from diarrheal diseases, it comes at a price of some unfavorable conditions or sanctions imposed on the country.^45^ Moreover, this benevolence from donor nations may come with a price tag of favors that may become a burden on the deteriorating economic situation of the country^45^ Furthermore, even after receiving the required number of dosages, the country might lack proper technology (reliance on sophisticated freezers) to store and transport the vaccines.^45^

In addition, the correct use of social media platforms can aid in reducing the incidence of diarrheal disease in children. These platforms have steadily gained popularity worldwide and Pakistan remains no exception to it. With this tremendous reach, social media can be used educationally by the health sector of government to control disease spread. From running educational and awareness campaigns to educating people about the severity of the disease, this tactic could reach more people than any other strategy. Discussions on how something considered as a casual stomach upset can transform into a deadly diarrhea, and how easily and quickly children can go into extreme states of dehydration that can cost them their lives. Furthermore, small educational videos can be run as ads on social media as government's public service message on basic first aid treatments to prevent progression to severity.^46^ This approach can also curb the challenge of delivering educational messages during COVID‐19 lockdowns, since face‐to‐face classes may be suspended.^47^

However, lack of monitoring on social media can prove to be harmful rather than beneficial. One prime example of this occurred in recent monsoon season in Pakistan where dengue was on the rise. False information regarding papaya leaf juice extract to be the cure of dengue as widely publicized on social media with no proof or scientific backup. The home remedy was readily adopted in many suffering households and resulted in several patients almost collapsing in hospitals due to severe diarrhea from the extract that caused extensive fluid.^48^ Therefore, correct usage and management of information spread on social media platforms is critical to achieve the desired goal of a healthier population. In addition, the use of complementary and alternative medicines (CAM) such as herbs, vitamins, and biological therapies are becoming increasingly popular and may provide benefit in boosting the immune system against diarrhea‐causing organisms.^49^

Diarrhea, one of the principal factors of child mortality in Pakistan, is a major threat to the accomplishment of millennium development goals in the country. Diarrhea is acquired chiefly through contaminated food and water, commonly by civilians that lack access to clean water and sanitation, such as those impoverished.^2^ The unwavering rise in the poverty rate demonstrates millions of Pakistan is being pushed into poverty every year.^20^ To make matters worse, over 2 million Pakistanis fell below the poverty line in 2020, possibly due to restrictions and lockdowns due to the COVID‐19 pandemic.^50^ Widespread poverty results in poor sanitation, limitations in receiving healthcare, and inadequate knowledge of common diseases, all contributing to the rise of diarrheal diseases.^51^

Among the provinces, Sindh leads both with the number of cases and number of deaths caused by diarrhea, with over 150,000 cases reported just in 2014.^52^ Most cases are reports from underdeveloped areas such as Tharparkar, Hyderabad, Mirpurkhas and other districts where there is lack of sanitation services such as functional filtration plants and poor infrastructure which lead to reckless incidents like mixing of sewage and water supply lines, making water extremely unfit for consumption. Sindh suffers the most in terms of water sanitation services due to lack of government investments.

Consumption of contaminated water remains a major reason for the deaths of children under 5 in Pakistan.^53^ Analysis of water supplies throughout the country found major impurities with microbes, arsenic, nitrates, and fecal contaminants.^54^, ^55^, ^56^ In Pakistan, water and sewerage pipes are placed alongside each other, allowing leakage of sewerage materials into sanitary water pipes.^57^ This provides a route for transmission of fecal‐oral pathogens such as *E. coli*, cholera, typhoid, and rotavirus. When sampling the contaminated water, the prevalence of *E. coli* was 51%, Enterobacter was 64%, and *G. lamblia* was 74%.^58^, ^59^, ^60^, ^61^ Waterborne diseases, stemming from consumption of unhealthy water, accounts for 80% of all diseases in Pakistan, especially diarrheal illnesses.^62^ This unsanitary condition is exacerbated by improper personal hygiene amongst children since most don't participate in frequent handwashing.

For every 1000 babies born in Pakistan in 2020, 65 don't make it to their 5th birthday.^63^ One major cause of infant mortality is inadequate breastfeeding. Breastfeeding is widely accepted as a vital protector against various harmful pathogens, especially for the first months of life. The American Academy of Pediatrics recommends breastfeeding for the first 6 months, and the most recent updates have extended the recommendation until the first 2 years of life.^64^ Antibodies against pathogens that the mother and baby have been exposed to are secreted through breastmilk and provide protection to the immature immune system of the baby. Most importantly, Immunoglobulin A coats the intestines of the infant, preventing the entrance of pathogens with fecal‐oral route, such as rotavirus.^24^ Moreover, a meta‐analysis of 18 publications supported the protective effects of exclusive breastfeeding against diarrhea.^65^

Despite its benefits, majority of Pakistani mothers do not indulge in this healthy practice. The repercussions are exhibited by research which shows acute gastroenteritis, caused by rotavirus, to be the major cause of death in Pakistani infants.^66^ For children who are not breastfed, another threat to their health is inadequate sterilization of bottles. For example, one Pakistani study showed the prevalence of the dysentery‐causing Campylobacter bacteria in 46.8% of bottle‐fed children, which was double the amount found in breastfed children.^67^ This pathogen is transmitted by a foodborne route, commonly transmitted through raw or contaminated milk, and the study found that breastfed infants were at a significantly lower risk of enteric infection.^67^ They concluded by stating consistent findings with other reports of reduced incidence of diarrhea amongst breastfed children.^67^ The recent surge of diarrheal cases in Karachi is believed to be due to using unsterilized water bottles or using contaminated water to make feeders, since most of the patients are between the ages of 3 months and 5 years, when bottle‐feeding is common.^15^ The use of boiled water for preparation of formula milk remains relatively low, at less than 32% in one study.^67^

Along with respiratory infections, diarrhea is one of the leading causes of antibiotic use in children in low and middle‐income countries such as Pakistan.^68^ Misusing antibiotics to cure diarrhea increases resistance to drugs by different pathogens (bacteria, viruses, or parasites alike), leading to lack of effectiveness and eradication. The lax practice of some healthcare professionals in prescribing unneeded antibiotics, as well as a general increase in self‐medication in Pakistan, is harboring a spike in drug resistant organisms, especially those that cause diarrhea.^69^

Globally, Pakistan is ranked third with the most unvaccinated children. The consequences of this shortcoming are apparent, as the country has the third‐highest burden of child mortality in the world.^9^ The EPI was launched in Pakistan in 1978, and provides protection from deadly yet preventable childhood diseases such as Tuberculosis, Diphtheria, Rotavirus, and Poliomyelitis.^9^ In Pakistan, rampant vaccine hesitancy and barriers to provide vaccinations have deprived about 3 million Pakistani children annually from acquiring a full course of childhood vaccinations, despite it being widely available. One major reason for vaccine hesitancy amongst parents was vaccine hesitancy, with one poll depicting 77% of parents expressing concerns regarding childhood vaccines.^9^ However, the true efficacy of the EPI is not apparent due to a lack of proper surveillance systems. One study found that children who have received the Rotavirus vaccine have not shown to have acquired any severe form of rotavirus gastroenteritis, and worldwide, the Rotavirus vaccination prevented 91% of rotavirus infections within the first year of life.^70^

To make matters worse, a common clinical presentation of COVID‐19 in children is diarrhea, which may present with or without respiratory symptoms.^71^ The clinical presentations of various diarrhea‐causing pathogens mimic COVID‐19 and may cause clinical misdiagnosis. Therefore, it is recommended to test for COVID‐19 in patients that are presenting with gastrointestinal symptoms.^72^ However, an inadequate testing capacity along with a surge in COVID‐19 cases daily will prove arduous in the correct diagnosis and treatment of patients, especially children, presenting with diarrhea.^29^, ^73^

In the past few years however, the prevalence of diarrhea has changed very little across Pakistan and management of the disease has brought a significant amount of reduction in childhood mortality. One study measured the prevalence of childhood diarrhea and pneumonia 15 years after introducing improved sanitation, safe drinking water, and engagement between healthcare workers and the community in a rural village of Pakistan. They reported a fourfold decrease in childhood mortality overall and a twofold decrease in morbidity due to diarrhea and pneumonia due to improvements in water quality, sanitation, and housing.^74^

The provision of safe drinking water is key to reduce childhood mortality due to diarrhea. Protection from pollutants, chlorination and boiling of water, distribution system integrity, and hygiene are vital practices to reduce mortality.^75^, ^76^ Boiling water is microbiologically effective in eradicating diarrhea‐causing organisms including vibrio cholerae, *G. lamblia*, *Cryptosporidium parvum*, rotavirus, and salmonella typhi.^77^ Mothers should be encouraged to use boiled water for preparation of formula milk to reduce the prevalence of diarrhea amongst bottle‐fed children.^67^ Approximately $442 million worth of funds were also approved by the World Bank in 2021 to enhance the sanitation services of Pakistan. The Punjab Rural Sustainable Water Supply and Sanitation Project aims to increase accessibility to clean water for more than six million rural residents who are deprived of it.^78^ Since the province of Sindh holds the highest record of childhood fatality due to diarrhea, it is the most important province to begin improving sanitation services.

Introduced by the WHO and UNICEF, the Integrated Management of Newborn and Childhood Illnesses (IMNCI) is a proven global strategy to reduce childhood mortality and morbidity and was adopted in Pakistan during the early 2000s. The IMNCI chart booklet mentions the plan of action when encountered by a sick child, and includes assessment, classification, and treatment.^79^ Implementation proved to be successful mainly for people in rural and underdeveloped areas where travelling to tertiary healthcare facilities is arduous, either due to poverty or lockdowns during the COVID‐19 era.^80^ Doctors, nurses, and other healthcare workers at basic health units in these underdeveloped areas were trained under the IMNCI program to effectively manage and treat common childhood diseases. However, implementation of IMNCI has been subpar in Pakistan, mainly due to the long training duration.^80^ A stronger approach to the widespread assimilation of IMNCI into all hospitals and rural areas must be implemented to reap the full benefits of this program. This will require clinical mentoring, adequate supplies and medication, as well as supportive supervision.^80^

To combat the surge in COVID‐19 cases and to prevent misdiagnosis, adequate provision of testing equipment as well as personal protective equipment are essential. In addition, healthcare workers must be adequately trained in offering breastfeeding counselling to mothers instead of opting to prescribe formula milk. Pakistan ranks at 18% for the early initiation of breastfeeding and lack of participation in breastfeeding has increased the number of stunted Pakistani children to 44%.^25^ Healthcare professionals must also practice calculated use of antibiotics to decrease the chances of drug resistance pathogens that cause diarrhea.

Among the successful treatment interventions of diarrhea, ORS tops the list.^81^ One study found that 72.1% of Pakistani mothers have used ORS for diarrhea, indicating its wide use in society.^82^ However, preparation of ORS requires accuracy, and is therefore misused by many.^83^ Hence, concerted awareness campaigns should be organized to educate parents to practice the correct use and method of utilizing ORS.^82^ Antimicrobials, zinc, and vitamin A supplementations have proven beneficial as well.^81^ However, many supplements are not widely available in pharmacies across the country.^83^ To alleviate this problem, UNICEF along with the Bill and Melinda Gates Foundation have invested over 12 million dollars for supplying medications and commodities and training staff for the treatment of childhood diarrhea and pneumonia. Districts from Sindh and Punjab were selected to have uniform representation.^12^ An evaluation 1 year later describes the project as successful, as 22,700 lady healthcare workers were trained in use of commodities in Sindh.^12^ The Punjab government intends to invest in it further to increase the project's span up to 10 more areas across the province.^83^

In low‐income countries, eating healthy is considered no less than a luxury. Vegetable and fruit prices have soared while high fat, sugary junk foods have become cheaper, compelling people to choose the unhealthy option Households in Pakistan spend about two‐thirds of their income on food compared with 13% in high income countries.^84^ Since an ounce of prevention is better than a pound of cure, the government should enhance the availability and affordability of fruits and vegetables to decrease the population's consumption of unhealthy food sources. Nutritional therapy such as including complex carbohydrates, yogurt, fruits, and vegetables should be popularized through campaigns to confer strong immunity.^81^ In addition, changes to the overall economy must be implemented by the introduction of jobs to boost the population's purchasing power since the primary barrier for not seeking hospital care was unaffordability (53%).^85^

Recently, EPI Pakistan has successfully added new vaccines and raised coverage to over 80% of population.^86^, ^87^ To enhance EPI services in Pakistan, offering regular EPI services throughout all existing health facilities, establishing an EPI center in all union councils, redistributing available vaccinators, and involving all trained manpower for vaccination will increase public access and compliance to the service and thus raise coverage significantly.^87^ The government must promote EPI awareness campaigns and advertisements regarding diarrhea's prevention and management to educate the masses and remove misinformation (Table 1).^86^

**Table 1:** Prevalence of diarrhea amongst Pakistani children in 2022

Month	Number of children affected	Province affected	Cause
April−May	2000	Punjab	Contaminated water
April	>100	Punjab	Poor personal hygiene
March	>100	Sindh	Food and water born^48^
April	114	Sindh, Baluchistan, Punjab	Contaminated water

## 5 LIMITATIONS

Adequate numbers of children affected by diarrheal illnesses and correct figures regarding the implementation of IMNCI and EPI were not available throughout the literature due to lack of a proper surveillance system.

## 6 CONCLUSION

Pakistani children are losing their lives daily due to preventable and treatable causes of diarrhea. The provision of clean drinking water is the most necessary step for prevention of disease. Healthcare workers must be sufficiently trained to offer quality counseling to encourage breastfeeding. In addition, the EPI program must be more adequately utilized by the masses throughout the country. The government must play an active role in raising awareness of vaccinations amongst society to remove fabrications that might prove deadly to children.

## AUTHOR CONTRIBUTIONS

**Zainab Syyeda Rahmat**: Writing – original draft; Writing – review and editing. **Amraha Zubair**: Writing – original draft. **Ikran Abdi**: Writing – original draft. **Narmil Humayun**: Writing – original draft. **Fatima Arshad**: Writing – original draft. **Mohammad Yasir Essar**: Conceptualization; Writing – review and editing.

## CONFLICT OF INTEREST

The authors declare no conflict of interest.

## TRANSPARENCY STATEMENT

The lead author Mohammad Yasir Essar affirms that this manuscript is an honest, accurate, and transparent account of the study being reported; that no important aspects of the study have been omitted; and that any discrepancies from the study as planned (and, if relevant, registered) have been explained.

## Data Availability

All data are available in the manuscript.

## References

[1] NIDDK . Definition & Facts for Diarrhea. Accessed July 14, 2022. https://www.niddk.nih.gov/health-information/digestive-diseases/diarrhea/definition-facts

[2] Diarrhoeal disease [Internet] . 2017. Accessed July 14, 2022. https://www.who.int/news-room/fact-sheets/detail/diarrhoeal-disease

[3] Fleisher RG , O'Ryan GMiguel , Levy J . Patient education: acute diarrhea in children (Beyond the Basics)—UpToDate [Internet]. 2022. Accessed July 14, 2022. https://www.uptodate.com/contents/acute-diarrhea-in-children-beyond-the-basics

[4] Diarrhea : common illness, global killer [Internet]. Accessed July 14, 2022. https://www.cdc.gov/healthywater/pdf/global/programs/globaldiarrhea508c.pdf

[5] Iddrisu I , Monteagudo‐Mera A , Poveda C , et al. Malnutrition and gut microbiota in children. Nutr. 2021;13:2727. https://www.mdpi.com/2072-6643/13/8/2727/htm 10.3390/nu13082727 PMC8401185 34444887

[6] Baqui AH , Ahmed T . Diarrhoea and malnutrition in children: replacing fluid and minerals, particularly zinc, remains vital. BMJ Br Med J [Internet]. 2006;332(7538):378. 16484244 10.1136/bmj.332.7538.378 PMC1370961

[7] Kahlown MA , Tahir MA , Rasheed H , Bhatti KP . Water quality status, national water quality monitoring programme. Fourth Tech ReportPCRWR. 2006;5.

[8] Population, total—Pakistan | Data [Internet] . Accessed July 2, 2022. https://data.worldbank.org/indicator/SP.POP.TOTL?locations=PK

[9] Saeed R , Hashmi I . Pakistan ranks third globally with the most unvaccinated children: is the impact of parental perception and attitude on immunization an essential contributing factor to an unsuccessful vaccination coverage? Cureus [Internet]. 2021;13(11):e19751. 34938628 10.7759/cureus.19751 PMC8684801

[10] Burki T . Childhood diarrhoea in Pakistan. Lancet Infect Dis. 2014;14(3):190‐191. http://www.thelancet.com/article/S1473309914700763/fulltext

[11] Daud MK , Nafees M , Ali S , et al. Drinking water quality status and contamination in Pakistan. BioMed Res Int. 2017;2017:1‐18. 10.1155/2017/7908183 PMC5573092 28884130

[12] UNICEF Pakistan [Internet] . Accelerating Policy Change, Translation and Implementation for Pneumonia and Diarrhea Commodities. Accessed July 14, 2022. https://www.unicef.org/pakistan/reports/accelerating-policy-change-translation-and-implementation-pneumonia-and-diarrhea

[13] World News . Diarrhoea outbreak hits Pakistan's Lahore, 2,000 children hospitalised since April 1. 2022. Accessed July 21, 2022. https://www.indiatoday.in/world/story/diarrhoea-outbreak-pakistan-lahore-children-hospitalised-1950831-2022-05-18

[14] Viral diarrhoea breaks out among children [Internet] . 2022. Accessed July 21, 2022. https://tribune.com.pk/story/2351611/viral-diarrhoea-breaks-out-among-children

[15] Diarrhoeal diseases rising sharply in Karachi as weather takes a turn ‐ Pakistan ‐ DAWN.COM [Internet] . 2022. Accessed July 21, 2022. https://www.dawn.com/news/1679863

[16] Cholera – Pakistan [Internet] . 2022. Accessed July 23, 2022. https://www.who.int/emergencies/disease-outbreak-news/item/2022-DON391

[17] Baloch SM . Pakistan town blames deadly cholera outbreak on government neglect | Global development | The Guardian [Internet]. 2022. Accessed July 23, 2022. https://www.theguardian.com/global-development/2022/may/23/pakistan-town-blames-deadly-cholera-outbreak-on-government-neglect

[18] Azadi NA , Ziapour A , Lebni JY , Irandoost SF , Abbas J , Chaboksavar F . The effect of education based on health belief model on promoting preventive behaviors of hypertensive disease in staff of the Iran University of Medical Sciences. Arch Public Health. 2021;79(1):69. 10.1186/s13690-021-00594-4 33952339 PMC8097917

[19] Toor IA , Sabihuddin Butt M . Socioeconomic and Environment Conditions and Diarrheal Disease Among Children in Pakistan.

[20] Development Bank A . Poverty in Pakistan: Issues, Causes, and Institutional Responses. 2002.

[21] Poverty in Pakistan rises to over 5% in 2020, estimates World Bank | Business Standard News [Internet]. 2021. Accessed July 17, 2022. https://www.business-standard.com/article/international/poverty-in-pakistan-rises-to-over-5-in-2020-estimates-world-bank-121062200084_1.html

[22] DAWN.COM . 53,000 children die of diarrhoea caused by contaminated water every year—Newspaper. 2018. Accessed July 14, 2022. https://www.dawn.com/news/1381735

[23] Patel HH . Water‐borne diseases [Internet]. Accessed July 15, 2022. https://www.news-medical.net/health/Water-Borne-Diseases.aspx

[24] Benefits of breastfeeding: for baby and mom [Internet] . Accessed July 20, 2022. https://my.clevelandclinic.org/health/articles/15274-the-benefits-of-breastfeeding-for-baby--for-mom

[25] WHO EMRO . Breastfeeding gives babies the best possible start in life and breastmilk works like a baby's first vaccine | Pakistan‐news | Pakistan [Internet]. 2017. Accessed July 19, 2022. http://www.emro.who.int/pak/pakistan-news/breastfeeding-gives-babies-the-best-possible-start-in-life-and-breastmilk-works-like-a-babys-first-vaccine.html

[26] Ge T , Abbas J , Ullah R , Abbas A , Sadiq I , Zhang R . Women's entrepreneurial contribution to family income: innovative technologies promote females' entrepreneurship amid COVID‐19 crisis. Front Psychol. 2022;13:282. 10.3389/fpsyg.2022.828040 PMC9004668 35422737

[27] Al Halbusi H , Al‐Sulaiti K , Abbas J , Al‐Sulaiti I . Assessing factors influencing technology adoption for online purchasing amid COVID‐19 in Qatar: moderating role of word of mouth. Front Environ Sci. 2022;10:1039.

[28] Geng J , Haq SU , Abbas J , et al. Survival in pandemic times: managing energy efficiency, food diversity, and sustainable practices of nutrient intake amid COVID‐19 crisis. Front Environ Sci. 2022;10:861.

[29] Pakistan's Covid‐19 positivity ratio reaches five‐month high [Internet] . 2022. Accessed July 14, 2022. https://www.thenews.com.pk/print/973259-pakistan-s-covid-19-positivity-ratio-reaches-five-month-high

[30] COVID‐19 Health Advisory Platform by Ministry of National Health Services Regulations and Coordination [Internet] . Accessed July 2, 2022. https://covid.gov.pk/stats/pakistan

[31] Farzadfar F , Naghavi M , Sepanlou SG , et al. Health system performance in Iran: a systematic analysis for the global burden of disease study 2019. Lancet. 2022;399(10335):1625‐1645. https://pubmed.ncbi.nlm.nih.gov/35397236/ 35397236 10.1016/S0140-6736(21)02751-3 PMC9023870

[32] Cork MA , Henry NJ , Watson S , et al. Mapping subnational HIV mortality in six Latin American countries with incomplete vital registration systems. BMC Med [Internet]. 2021;19(1):1‐25. https://bmcmedicine.biomedcentral.com/articles/10.1186/s12916-020-01876-4 33413343 10.1186/s12916-020-01876-4 PMC7791645

[33] Schmidt CA , Cromwell EA , Hill E , et al. The prevalence of onchocerciasis in Africa and Yemen, 2000‐2018: a geospatial analysis. BMC Med. 2022;20(1):293. 10.1186/s12916-022-02486-y 36068517 PMC9449300

[34] Li X , Abbas J , Dongling W , Baig NUA , Zhang R . From cultural tourism to social entrepreneurship: role of social value creation for environmental sustainability. Front Psychol. 2022;13:3261. 10.3389/fpsyg.2022.925768 PMC9332340 35911048

[35] NeJhaddadgar N , Ziapour A , Zakkipour G , Abbas J , Abolfathi M , Shabani M . Effectiveness of telephone‐based screening and triage during COVID‐19 outbreak in the promoted primary healthcare system: a case study in ardabil province, Iran. J Public Health (Bangkok). 2022;30(5):1301‐1306. https://link.springer.com/article/10.1007/s10389-020-01407-8 10.1007/s10389-020-01407-8 PMC7665795 33224715

[36] Aqeel M , Abbas J , Shuja KH , et al. The influence of illness perception, anxiety and depression disorders on students mental health during COVID‐19 outbreak in Pakistan: a web‐based cross‐sectional survey. Int J Hum Rights Healthc. 2022;15(1):17‐30.

[37] Abbas J . The impact of coronavirus (SARS‐CoV2) epidemic on individuals mental health: the protective measures of Pakistan in managing and sustaining transmissible disease. Psychiatr Danubina. 2020;32(3‐4):472‐477. https://pubmed.ncbi.nlm.nih.gov/33370755/ 10.24869/psyd.2020.472 33370755

[38] Soroush A , Ziapour A , Abbas J , et al. Effects of group logotherapy training on Self‐Esteem, communication skills, and impact of event Scale‐Revised (IES‐R) in older adults. Ageing Int. 2021;47:758‐778. 10.1007/s12126-021-09458-2

[39] Venkatesan MM , van de Verg LL . Combination vaccines against diarrheal diseases. 2015. Accessed November 15, 2022. 10.4161/216455152014986984 https://www.tandfonline.com/doi/abs/10.4161/21645515.2014.986984 PMC4517455 25891647

[40] Yao J , Ziapour A , Abbas J , Toraji R , NeJhaddadgar N . Assessing puberty‐related health needs among 10–15‐year‐old boys: A cross‐sectional study approach. Archives de Pédiatrie. 2022;29(4):307‐311. 35292195 10.1016/j.arcped.2021.11.018

[41] Sheikh M , Jehan F . A look at vaccine equity from Pakistan | Think Global Health [Internet]. 2021. Accessed November 15, 2022. https://www.thinkglobalhealth.org/article/look-vaccine-equity-pakistan

[42] Jiakui C , Abbas J , Najam H , Liu J , Abbas J . Green technological innovation, Green finance, and financial development and their role in Green total factor productivity: empirical insights from China. J Clean Prod. 2023;382:135131. https://linkinghub.elsevier.com/retrieve/pii/S0959652622047059

[43] Zhuang D , Abbas J , Al‐Sulaiti K , Fahlevi M , Aljuaid M , Saniuk S . Land‐use and food security in energy transition: role of food supply. Front Sustai Food Syst. 2022;6:510.

[44] GT investigates: rich countries hoarding vaccines in disregard of poorer regions breathes life into new variants, worsens economic disparity—Global Times [Internet] . 2022. Accessed November 15, 2022. https://www.globaltimes.cn/page/202201/1246196.shtml

[45] Su Z , McDonnell D , Li X , et al. COVID‐19 vaccine Donations—vaccine empathy or vaccine diplomacy? A narrative literature review. Vaccines. 2021;9:1024. https://www.mdpi.com/2076-393X/9/9/1024/htm 34579261 10.3390/vaccines9091024 PMC8470866

[46] Yu S , Abbas J , Draghici A , Negulescu OH , Ain NU . Social media application as a new paradigm for business communication: the role of COVID‐19 knowledge, social distancing, and preventive attitudes. Front Psychol. 2022;13:2228. 10.3389/fpsyg.2022.903082 PMC9160995 35664180

[47] Rahmat T , Raza S , Zahid H , Abbas J , Mohd Sobri F , Sidiki S . Nexus between integrating technology readiness 2.0 index and students’ e‐library services adoption amid the COVID‐19 challenges: implications based on the theory of planned behavior. J Educ Health Promot [Internet] . 2022;11(1). https://pubmed.ncbi.nlm.nih.gov/35372596/ 10.4103/jehp.jehp_508_21 PMC8974977 35372596

[48] Bhatti WM . Experts warn of outbreak of gastroenteritis in Karachi, rest of Sindh [Internet]. 2022. Accessed July 23, 2022. https://www.thenews.com.pk/print/943227-experts-warn-of-outbreak-of-gastroenteritis-in-karachi-rest-of-sindh

[49] Zeidabadi S , Abbas J , Mangolian Shahrbabaki P , Dehghan M . The effect of foot reflexology on the quality of sexual life in hemodialysis patients: a randomized controlled clinical trial. Sex Disabil. 2022;40(3):567‐581.

[50] Poverty in Pakistan rises to over 5% in 2020, estimates World Bank | Business Standard News [Internet] . Accessed July 2, 2022. https://www.business-standard.com/article/international/poverty-in-pakistan-rises-to-over-5-in-2020-estimates-world-bank-121062200084_1.html

[51] Bilal W , Qamar K , Abbas S , Siddiqui A , Essar MY . Infectious diseases surveillance in Pakistan: challenges, efforts, and recommendations. Ann Med Surg. 2022;78:103838. 10.1016/j.amsu.2022.103838 PMC9207098 35734665

[52] Over 150,000 diarrhoea cases reported in Sindh last year ‐ Pakistan | ReliefWeb [Internet] . 2015. Accessed July 22, 2022. https://reliefweb.int/report/pakistan/over-150000-diarrhoea-cases-reported-sindh-last-year

[53] Murtaza F , Muzaffar M , Mustafa T , Anwer J . Water and sanitation risk exposure in children under‐five in Pakistan. J Fam Comm Med. 2021;28:103. 10.4103/jfcm.jfcm_149_21 PMC8213103 34194274

[54] Shoaib M , Aziz S , Usman M , Rehman A , Zafar MM , Ilyas M . Prevalence of Pathogenic Microorganisms in Drinking Water of Rawalpindi and Islamabad. 2016.

[55] Awan A , Nasir MS , Shaukat I , Anwar S , Ayub I . Impact of samanduri drain on water resources of faisalabad. Adv Environ Biol. 2016;10:155‐160.

[56] Soomro Z , Khokhar W , Hussain M , Hussain I . Drinking water quality challenges in Pakistan. 2022.

[57] Patoli AA , Patoli BB , Mehraj V . High prevalence of multi‐drug resistant Escherichia coli in drinking water samples from Hyderabad. Gomal J Med Sci. 2010;8.

[58] Jabeen S , Mahmood Q , Tariq S , Nawab B , Elahi N . Health impact caused by poor water and sanitation in district abbottabad. J Ayub Med Coll Abbott. 2011;23:47‐50. 22830145

[59] Baqai R . Water contamination and its related diseases. 3138465

[60] Apanga PA , Ahmed J , Tanner W , et al. Carbapenem‐resistant enterobacteriaceae in sink drains of 40 healthcare facilities in sindh, Pakistan: a cross‐sectional study. PLoS One. 2022;17(2):e0263297. 35113948 10.1371/journal.pone.0263297 PMC8812900

[61] khan SA , Ayaz S , Khan S , Anees M , Khan AM , Khan I . Prevalence of *giardia lamblia* in different water sources of district nowshehra, khyber pakhtunkhwa Pakistan. Int J Adv Res & Technol. 2012;1:125‐132.

[62] Ahmed T , Zounemat‐Kermani M , Scholz M . Climate change, water quality and Water‐Related challenges: a review with focus on Pakistan. Int J Environ Res Public Health. 2020;17:8518. 33212957 10.3390/ijerph17228518 PMC7698392

[63] Poverty: Pakistan | Asian Development Bank [Internet] . Accessed July 2, 2022. https://www.adb.org/countries/pakistan/poverty

[64] Lucia CA , Hartshorn J . The benefits of breastfeeding for parent and baby [Internet]. 2021. Accessed July 20, 2022. https://www.parents.com/baby/breastfeeding/basics/the-benefits-of-breastfeeding/

[65] Lamberti LM , Fischer Walker CL , Noiman A , Victora C , Black RE . Breastfeeding and the risk for diarrhea morbidity and mortality. BMC Public Health. 2011;11(suppl 3):S15. 10.1186/1471-2458-11-S3-S15 PMC3231888 21501432

[66] Ali S , Khan S , Khan SN , et al. Molecular detection and prevalence of Rotavirus with acute gastroenteritis among the children of rural and urban areas. Brazilian J Biol [Internet]. 2021;83. http://www.scielo.br/j/bjb/a/XfQrDh3grqZX6kdNb88WqGS/?lang=en 10.1590/1519-6984.244365 34932615

[67] Shahid M , Amjad M , Kazmi SU . Breast feeding may protect children against Campylobacter jejuni associated diarrhoea—PubMed [Internet]. Accessed July 23, 2022. https://pubmed.ncbi.nlm.nih.gov/12346517/ 12346517

[68] Defeat diarrhea and help stop drug resistance, too [Internet] . 2020. Accessed July 22, 2022. https://www.defeatdd.org/blog/defeat-diarrhea-and-help-stop-drug-resistance-too

[69] Atif M , Asghar S , Mushtaq I , et al. What drives inappropriate use of antibiotics? A mixed methods study from bahawalpur, Pakistan. Infect Drug Resist [Internet]. 2019;12:687‐699. https://pubmed.ncbi.nlm.nih.gov/30988635/ 30988635 10.2147/IDR.S189114 PMC6440533

[70] Hussain A . Rotavirus vaccine demand in Pakistan. Iran J Publ Health. 2016;45(12):1658‐1659. PMC5207109 28053934

[71] Rathore V , Galhotra A , Pal R , Sahu KK . COVID‐19 pandemic and children: a review. J Pediatr Pharmacol Ther. 2020;25(7):574‐585. https://pubmed.ncbi.nlm.nih.gov/33041712/ 33041712 10.5863/1551-6776-25.7.574 PMC7541032

[72] Sukharani N , Kumar J , Makheja K , et al. Gastrointestinal manifestation of COVID‐19 in hospitalized patients. 2021. 10.7759/cureus.18024 PMC8520409 34667695

[73] Jamal S , Aijaz J , Shah N , et al. COVID‐19 testing crisis management through a public‐private partnership in sindh, Pakistan. Glob Heal Sci Pract [Internet]. 2022;10(1). https://www.ghspjournal.org/content/10/1/e2100308 10.9745/GHSP-D-21-00308 PMC8885349 35294380

[74] Hansen CL , McCormick BJJ , Azam SI , et al. Substantial and sustained reduction in under‐5 mortality, diarrhea, and pneumonia in oshikhandass, Pakistan: evidence from two longitudinal cohort studies 15 years apart. BMC Public Health. 2020;20(1):759. 10.1186/s12889-020-08847-7 32448276 PMC7245818

[75] Oleribe OO , Momoh J , Uzochukwu BSC , et al. Identifying key challenges facing healthcare systems in Africa and potential solutions. Int J Gen Med [Internet]. 2019;12:395. 31819592 10.2147/IJGM.S223882 PMC6844097

[76] Rabbani F , Zahidie A . Recent strategies to improve community case management of diarrhea among children under five in developing countries. Diarrhea Treat [Internet]. 2016. https://ecommons.aku.edu/pakistan_fhs_mc_chs_chs/204

[77] Cohen A , Colford JM . Effects of boiling drinking water on diarrhea and Pathogen‐Specific infections in low‐ and middle‐income countries: a systematic review and meta‐analysis. Am J Trop Med Hyg. 2017;97(5):1362‐1377. https://www.ajtmh.org/view/journals/tpmd/97/5/article-p1362.xml 29016318 10.4269/ajtmh.17-0190 PMC5817760

[78] Pakistan to invest in water and sanitation services to boost health and climate resilience in Punjab province [Internet] . 2021. Accessed July 22, 2022. https://www.worldbank.org/en/news/press-release/2021/06/18/pakistan-to-invest-in-water-and-sanitation-services-to-boost-health-and-climate-resilience-in-punjab-province

[79] Integrated management of neonatal and childhood illness‐chart booklet [Internet] . Accessed July 23, 2022. https://phkh.nhsrc.pk/sites/default/files/2020-02/IMNCIChartbookletPakistan2017.pdf

[80] WHO EMRO | Child health in Pakistan—turning a new page in IMNCI implementation | Pakistan‐events | Pakistan [Internet] . 2017. Accessed July 23, 2022. http://www.emro.who.int/pak/pakistan-events/child-health-in-pakistan-turning-a-new-page-in-imnci-implementation.html

[81] King CK , Glass R , Bresee JS , Duggan C . Managing acute gastroenteritis among children: oral rehydration, maintenance, and nutritional therapy [Internet]. 2003. Accessed July 22, 2022. https://www.cdc.gov/mmwr/preview/mmwrhtml/rr5216a1.htm 14627948

[82] Ahmed A , Malik IA , Iqbal M , et al. The use of ORS (Nimkol) in management of childhood diarrhoea by mothers in the suburbs of Rawalpindi‐Islamabad ‐ PubMed [Internet]. Accessed July 22, 2022. https://pubmed.ncbi.nlm.nih.gov/2126298/ 2126298

[83] Shahryar F . New commodities for management of pneumonia and diarrhea make a difference | UNICEF Pakistan [Internet]. 2018. Accessed July 23, 2022. https://www.unicef.org/pakistan/stories/new-commodities-management-pneumonia-and-diarrhea-make-difference

[84] Miller V , Yusuf S , Chow CK , et al. Availability, affordability, and consumption of fruits and vegetables in 18 countries across income levels: findings from the prospective urban rural epidemiology (PURE) study. Lancet Glo Health. 2016;4(10):e695‐e703. https://pubmed.ncbi.nlm.nih.gov/27567348/ 10.1016/S2214-109X(16)30186-3 27567348

[85] Rheingans R , Kukla M , Faruque ASG , et al. Determinants of household costs associated with childhood diarrhea in 3 south asian settings. Clin Infect Dis. 2012;55(suppl 4):S327‐S335. https://academic.oup.com/cid/article/55/suppl_4/S327/326853 23169945 10.1093/cid/cis764 PMC3502314

[86] Khan A , Khan S , Ullah I , et al. Evaluation of immunization coverage in the rural area of peshawar, khyber pakhtunkhwa. Cureus [Internet]. 2019;11(1):e3992. https://www.cureus.com/articles/11724-evaluation-of-immunization-coverage-in-the-rural-area-of-peshawar-khyber-pakhtunkhwa 30972271 10.7759/cureus.3992 PMC6443532

[87] Hasan Q , Bosan AH , Bile KM . A review of EPI progress in Pakistan towards achieving coverage targets: present situation and the way forward—PubMed [Internet]. 2010. Accessed July 22, 2022. https://pubmed.ncbi.nlm.nih.gov/21495586/ 21495586

